# The miR-223 host non-coding transcript linc-223 induces IRF4 expression in acute myeloid leukemia by acting as a competing endogenous RNA

**DOI:** 10.18632/oncotarget.11165

**Published:** 2016-08-09

**Authors:** Arianna Mangiavacchi, Melissa Sorci, Silvia Masciarelli, Simone Larivera, Ivano Legnini, Ilaria Iosue, Irene Bozzoni, Francesco Fazi, Alessandro Fatica

**Affiliations:** ^1^ Department of Biology and Biotechnology “C. Darwiny”, Sapienza University of Rome, Rome, 00185, Italy; ^2^ Department of Anatomical, Histological, Forensic and Orthopaedic Sciences, Sapienza University of Rome, Rome, 00185, Italy; ^3^ Center for Life Nano Science@Sapienza, Istituto Italiano di Tecnologia, Rome, 00161, Italy; ^4^ Institute Pasteur Fondazione Cenci-Bolognetti, Sapienza University of Rome, Rome, 00185, Italy; ^5^ Present address: KAUST Environmental Epigenetics Research Program, Biological Environmental Sciences and Engineering Division, King Abdullah University of Science and Technology, Thuwal 23955-6900, Saudi Arabia

**Keywords:** long non-coding RNA, miR-125, acute myeloid leukemia, IRF4

## Abstract

Alterations in genetic programs required for terminal myeloid differentiation and aberrant proliferation characterize acute myeloid leukemia (AML) cells. Here, we identify the host transcript of miR-223, linc-223, as a novel functional long non-coding RNA (lncRNA) in AML. We show that from the primary nuclear transcript, the alternative production of miR-223 and linc-223 is finely regulated during monocytic differentiation. Moreover, linc-223 expression inhibits cell cycle progression and promotes monocytic differentiation of AML cells. We also demonstrate that endogenous linc-223 localizes in the cytoplasm and acts as a competing endogenous RNA for miR-125-5p, an oncogenic microRNA in leukemia. In particular, we show that linc-223 directly binds to miR-125-5p and that its knockdown increases the repressing activity of miR-125-5p resulting in the downregulation of its target interferon regulatory factor 4 (IRF4), which it was previously shown to inhibit the oncogenic activity of miR-125-5p *in vivo*. Furthermore, data from primary AML samples show significant downregulation of linc-223 in different AML subtypes. Therein, these findings indicate that the newly identified lncRNA linc-223 may have an important role in myeloid differentiation and leukemogenesis, at least in part, by cross-talking with IRF4 mRNA.

## INTRODUCTION

It is becoming increasingly apparent the relevance of non-coding RNAs (ncRNAs) in the genesis and progression of human cancer. At present, the majority of the studies on ncRNAs have mainly focused on microRNAs (miRNAs) expression and function. However, genome-wide studies have now shown that the mammalian genome is largely transcribed and that a relevant percentage of this transcription produces the heterogeneous class of long-ncRNAs (lncRNAs). Overall, the human genome originates more than ten thousands lncRNAs that, similarly to miRNAs, show tissue- and developmental stage- specific expression. The repertoire of lncRNAs involved in gene expression regulation is growing fast, as there is now evidence that lncRNAs participate in multiple networks controlling development and disease [1].

LncRNAs are emerging as key regulators of the development and function of the hematopoietic system [2]. Indeed, several lncRNAs are highly expressed in specific hematopoietic lineages and manipulation of their levels has been correlated with changes in cellular properties or differentiation [2]. However, many of them are still not functionally characterized.

Acute myeloid leukemia (AML) is characterized by genetic and epigenetic alterations in progenitor cells that produce complete or partial blockage at different stages of myeloid differentiation and uncontrolled proliferation [3]. Studying the functional interactions between genes that control the correct balance between cell proliferation and differentiation is critical to understand how their deregulated expression may contribute to leukemogenesis. In this context, lncRNAs might play an important role in the molecular pathogenesis of leukemia interfering with pathways essential for hematopoietic differentiation.

One of the first miRNAs to be functionally characterized in normal and pathological myelopoiesis is miR-223 [4–6]. MiR-223 is preferentially expressed in myeloid cells and is induced during retinoic acid (RA)-mediated granulocytic differentiation of AML cells, at least in part, through the transcription factors C/EBPa and PU.1 [6–8]. MiR-223 is encoded in the third exon of what was previously defined a pri-miRNA transcript [7]. In this study we show that instead a spliced and polyadenylated non-coding RNA accumulates in the cytoplasm as an unprocessed species still containing the pre-miR-223 sequence (here referred to as linc-223). Different studies showed functional association between miRNAs and their host genes, both for coding [9–11] and non-coding host transcripts [12, 13].

Here, we show that linc-223 is a cytoplasmatic RNA that is induced and stably accumulated during differentiation of AML cells and human cord blood CD34^+^ progenitor cells. Through modulation of linc-223 levels, we demonstrated that linc-223 expression inhibits cell proliferation and stimulates differentiation of AML cells. We showed that linc-223 acts, at least in part, by cross talking with the tumor suppressor IRF4 mRNA through competition for the binding of the oncogenic miR-125-5p. Furthermore, we found that linc-223 expression is downregulated in primary AML.

## RESULTS

### Linc-223 expression is associated with myeloid differentiation

**Figure 1:**
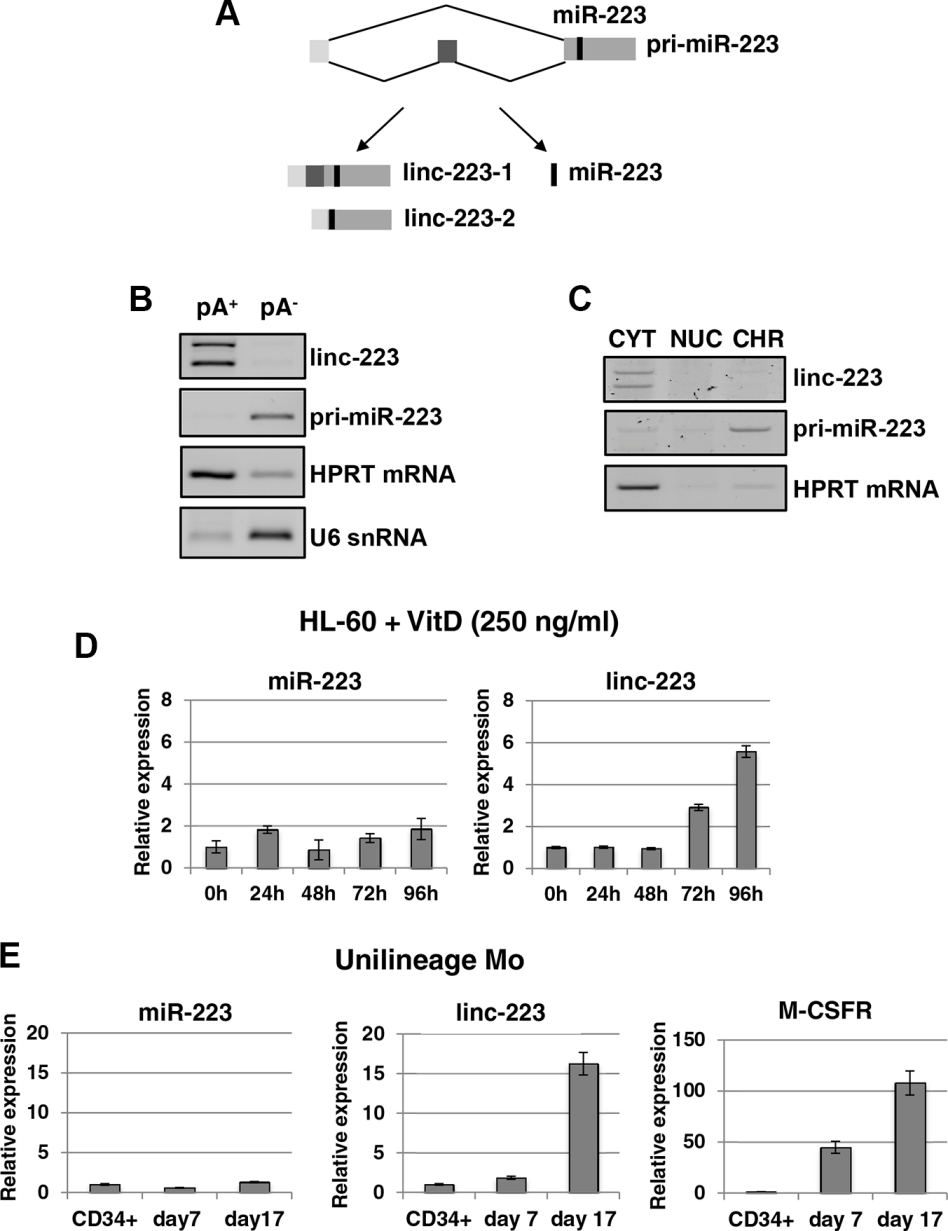
Linc-223 is a cytoplasmic lncRNA induced in monocytopoiesis (**A**) Schematic representation of miR-223 primary transcripts (prim-miR-223), which can produce two lncRNA isoforms (linc-223-1 and linc-223-2) and miR-223. (**B**) RT-PCR for linc-223, unspliced pri-miR-223, HPRT mRNA and U6 snRNA from polyadenylated (pA+) and nonpolyadenylated (pA-) RNA purified from HL-60 cells. (**C**). RT-PCR for linc-223, unspliced pri-miR-223 and HPRT mRNA from RNA isolated from cytoplasmic (CYT), nuclear (NUC), and chromatin (CHR) fractions of HL-60 cells. (**D**) qPCR analysis of miR-223 and linc-223 levels during VitD3-induced monocytic differentiation of HL-60 cell line. Values were normalized for U6 and HPRT mRNA expression, respectively. (**E**) qPCR analysis of miR-223, linc-223 and MCSFr levels during MCSF-induced monocytic differentiation of human CD34+ progenitor cells. Values were normalized for U6 (miR-223) and HPRT mRNA (linc-223 and MCSFr) expression. The histograms represent the means ± S.E.M. from triplicates.

AML cell lines are widely used to study the block of differentiation in haematopoiesis and represent a suitable *in vitro* model for AML. By using the HL-60 cell line, which can differentiate either into granulocytes or monocytes by treatment with all-trans retinoic acid (ATRA) or 1,25-dihydroxy-vitamin D3 (VitD3), respectively, we found that the miR-223 host transcript [7] accumulates in the cytoplasm as a spliced RNA still containing the pre-miR-223 sequence (Figure 1A). This RNA, which is devoid of any coding potential (Supplementary Figure S1), has been named linc-223. Two splicing variants were found, which differ by exon 2 inclusion, and which are here referred to as linc-223-1 (1-2-3 exons) and linc-223-2 (1-3 exons). RT-PCR analysis indicated that linc-223 isoforms are polyadenylated and are localized in the cytoplasm (Figures 1B and 1C). Moreover, using primer sets specific for the two linc-223 isoforms, we showed by real-time PCR (qRT-PCR) that linc-223 was significantly induced during VitD3-mediated monocytic differentiation without a tight correlated induction of miR-223 expression (Figure 1D), suggesting different regulatory mechanisms. Linc-223 expression was also analyzed during monocytic differentiation of human cord blood CD34^+^ progenitor cells using a couple of oligonucleotides that recognises both isoforms (Figure 1E). Also in this case, linc-223 expression increased during differentiation without concomitant induction of miR-223. High levels of linc-223 expression were also found in normal mature myeloid cells purified from peripheral blood compared to CD34+ progenitors (Figure 7). As previously reported [7, 14], we also detected increased linc-223 in ATRA-mediated granulocytic differentiation (Supplementary Figure S2). However, in this case it correlates with an increase of miR-223 levels [6, 7, 14]. Therein, these data indicate that from the primary nuclear transcript, the alternative production of miR-223 and linc-223 is finely regulated during monocytic differentiation, suggesting that along this lineage linc-223 may play a specific function.

### Linc-223 inhibits proliferation and stimulates monocytic differentiation of AML cells

**Figure 2:**
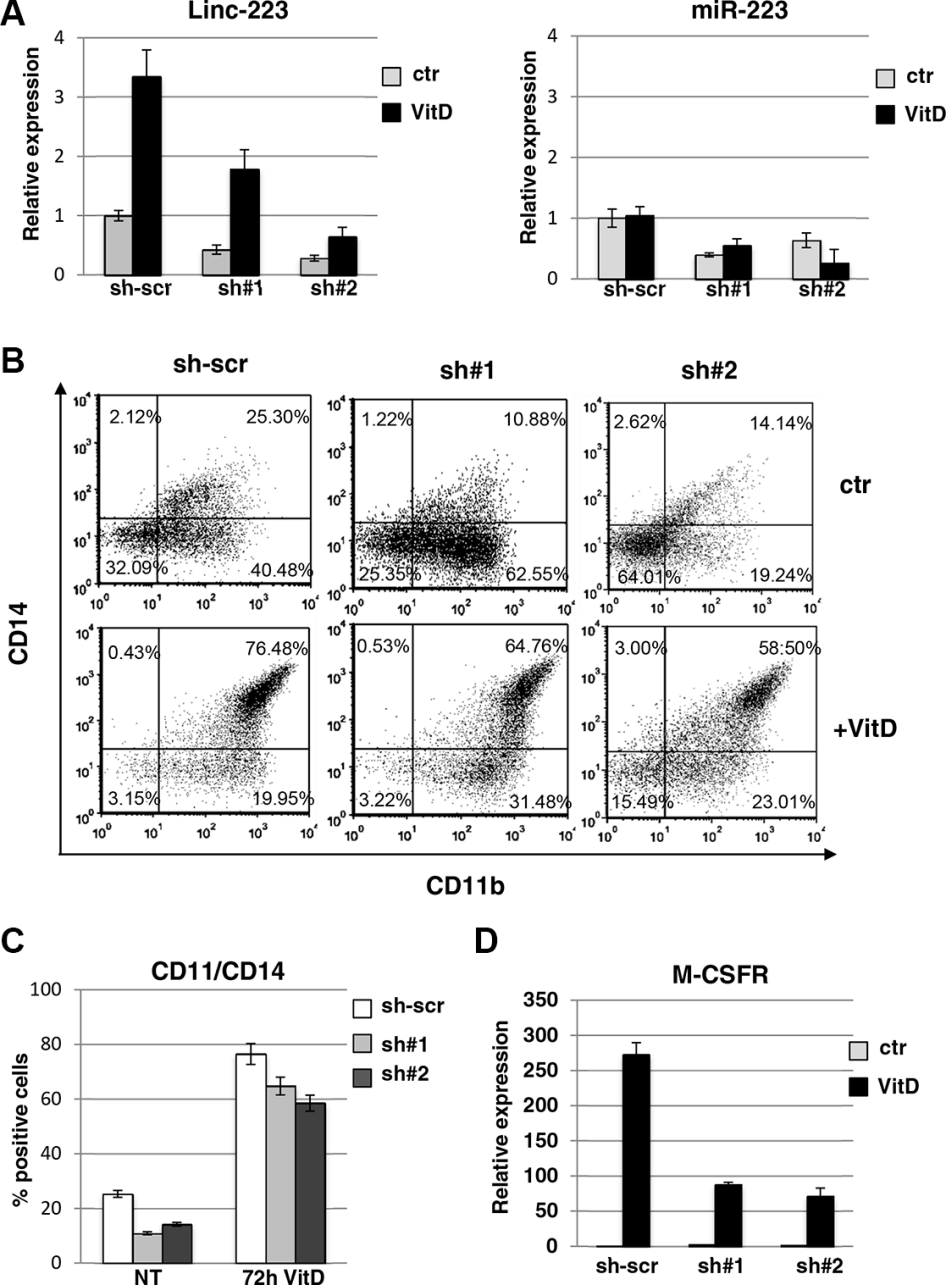
Lin-223 knockdown inhibits monocytic differentiation (**A**) qPCR analysis of miR-223 and linc-223 levels upon knockdown of linc-223 by two different lentiviral constructs expressing shRNAs (sh#1 and sh#2). Lentivirus expressing a scramble shRNA (sh-scr) was utilized as control. Values were normalized for U6 (miR-223) and HPRT mRNA (linc-223) expression. The histograms represent the means ± S.E.M. from triplicates. (**B**) FACS analysis of CD11b and CD14 positive cells. Data presented are from one representative experiment. (**C**) The histogram represents the percentage of CD11b/CD14 positive cells before and after VitD3 treatment in HL-60 cell expressing scramble shRNA or shRNAs againsts linc-223 (see panel A). (**D**) qPCR analysis of MCSFr levels in HL-60 cell expressing scramble shRNA or shRNAs againsts linc-223. Values were normalized for HPRT mRNA expression. The histograms represent the means ± S.E.M. from triplicates.

In order to assess the role of linc-223, we utilized shRNAs targeting linc-223 to down-regulate its expression levels. HL-60 cells were infected with two different lentiviral vectors expressing shRNAs against both linc-223 isoforms (sh#1), linc-223-1 (sh#2) or scramble shRNA (sh-scr) and differentiation was evaluated by analyzing the immunophenotype and induction of specific molecular markers. Cells infected with either sh#1 and sh#2 exhibited a significant decrease in linc-223 levels before and after VitD treatment (Figure 2A). Notably, a reduction of cells expressing the surface markers CD14 and CD11b, which are upregulated during myelomonocytic differentiation, was observed upon depletion of linc-223 (Figures 2B and 2C). Moreover, in line with these results, reduced expression levels of M-CSFr, a marker of monocytic differentiation, was also detected (Figures 2D and Supplementary Figure S3). The shRNAs against linc-223 produced also a decrease in miR-223 expression levels (Figure 2A). To exclude the involvement of miR-223 in monocytic differentiation following experiments were designed to specifically analyze linc-223 function.

**Figure 3:**
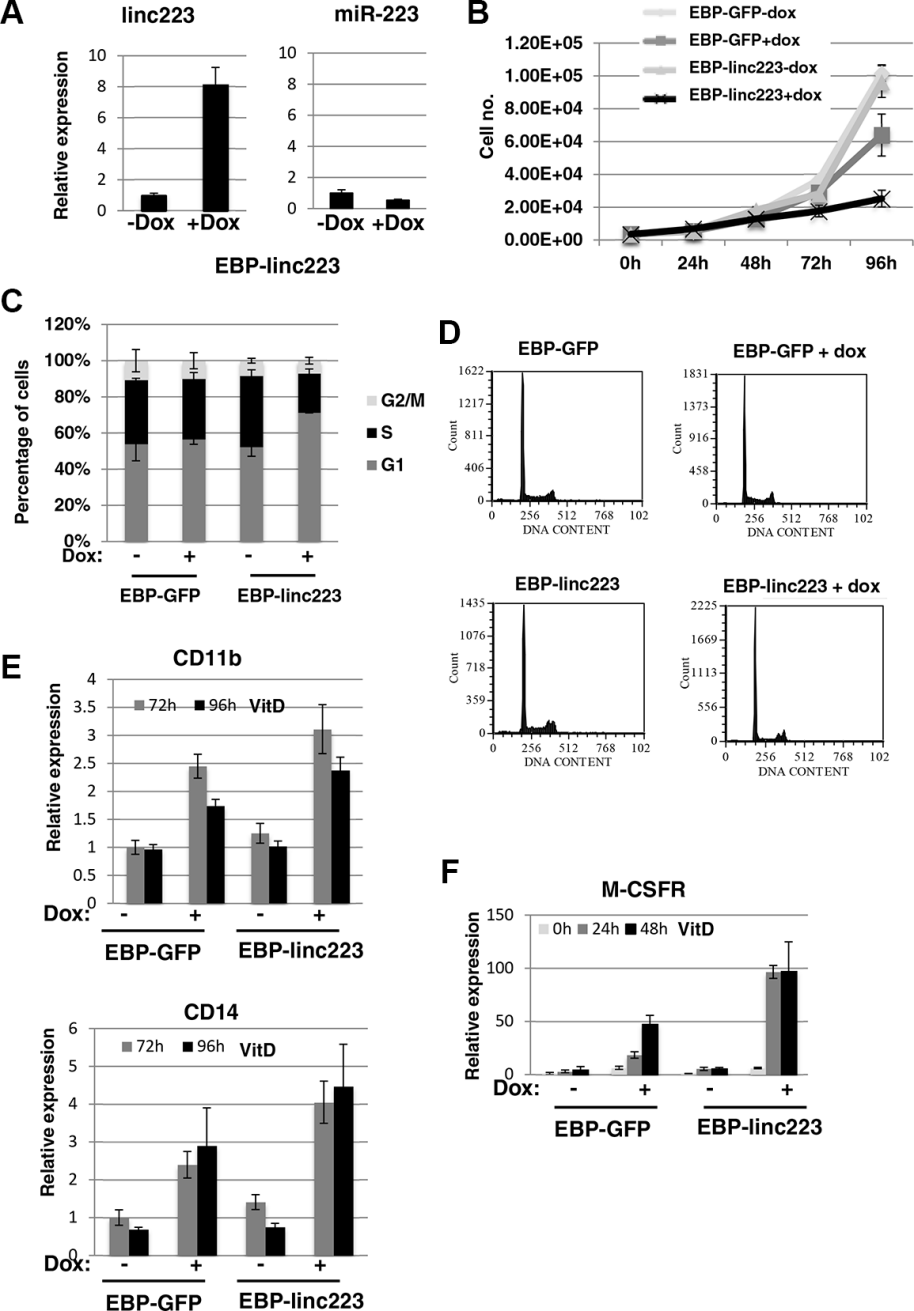
Ectopic expression of linc-223 inhibits proliferation and stimulates differentiation (**A**) Analysis of miR-223 and linc-223 levels in HL-60 cells containing linc-223 expression cassette (EBP-linc223) before (-dox) and after induction (+dox) with Doxycycline. Level of expression was measured by qRT-PCR relative to U6 snRNA for miR-223 and HPRT mRNA for linc-223. Error bars represent S.E.M. from three independent experiments. (**B**) Growth curve of HL-60 cells containing GFP (EBP-GFP) and linc-223 (EBP-linc223) expression cassette, respectively, before (-dox) and after induction (+dox) with Doxycycline. (**C**) Cell cycle distribution of cells with EBP-GFP and EBP-linc223 expression cassette. (**D**) Representative cell cycle analysis of (C). (**E**) qPCR analysis of CD11b and CD14 upon VitD3 treatment in HL-60 cells expressing GFP or linc223. (**F**) qPCR analysis of MCSFr levels in HL-60 cells expressing GFP or linc-223. Error bars represent S.E.M. from three independent experiments.

HL-60 cells were stably transformed with a PiggyBac transposon system [15, 16] carrying Tet-inducible linc-223 (EBP-linc223) and GFP (EBP-GFP), respectively. A linc-223 derivative deleted for the region required for Drosha cleavage was raised in order to specifically obtain linc-223 expression without any miR-223 contribution. Notably, the overexpression levels of linc-223 after doxycyclin (dox) induction are comparable with those obtained by VitD treatment (compare Figures 1D and 3A). Moreover, miR-223 expression levels are not increased by EBP-linc223 (Figure 3A). In AML the terminal differentiation block is strictly related to aberrant proliferation (3). Thus, we analyzed the effect of linc-223 on cellular proliferation and cell cycle. Ectopic expression of linc-223, but not of control GFP, greatly decreased the proliferation rate of HL-60 (Figure 3B). Moreover, cell cycle analysis showed that linc-223 expression increased the percentage of cells in G_1_ arrest and decreased the number of cells in S phase (Figures 3C and 3D). These data indicated that increased linc-223 levels are sufficient to inhibit proliferation of AML cells. We next analyzed the ability of VitD-treated HL-60 cells carrying the EBP-linc223 or the control EBP-GFP expression cassettes to undergo monocytic differentiation. Strong increase of cells expressing the surface markers CD14 and CD11b (Figure 3E) and of the marker of monocytic differentiation M-CSFr (Figure 3F) was indeed observed upon induction of linc-223 and treatment with suboptimal doses of VitD (25 ng/ml). Altogether, these results indicate that linc-223 stimulated differentiation of AML cells while diminishing their proliferation rate.

### Linc-223 cross-talks with miR-125-5p and IRF4 mRNA

**Figure 4:**
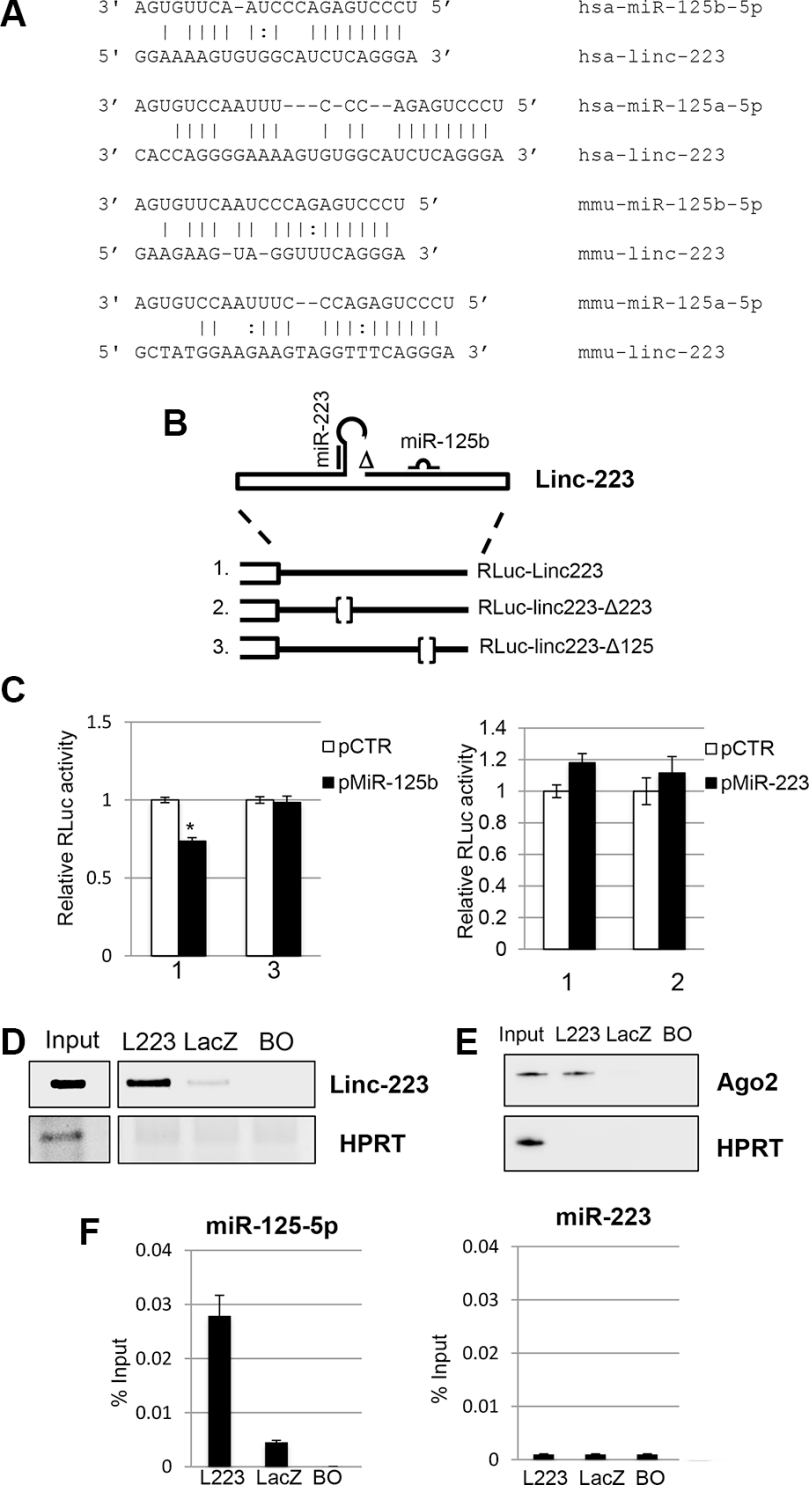
Linc-223 interacts with miR-125b (**A**) Base-pairing between miR-125-5p family members and linc-223 in human and mouse. (**B**) Schematic representation of linc-223 ΔDrosha constructs wild type (RLuc-Linc223) and mutant derivatives devoid of miR-223 (RLuc-linc223-Δ223) or miR-125b (RLuc-linc223-Δ125) binding sites used in the luciferase assays. (**C**) HL-60 cells were electroporated with linc223 reporters together with plasmids expressing miR-125b-5p (pMiR-125b) or miR-223 (pmiR-223) or with a control vector (pCTR). FF luciferase values were normalized to RL luciferase reading. Data, derived from five independent experiments, are shown with respect to RLuc control vector set to a value of 1. Data are shown as mean ± SD. Expression differences were statistically analyzed; *p* < 0.05 = * (**D**) Biotinylated antisense DNA oligonucleotides complementary to linc-223 (L223) were incubated with cytoplasmic extracts of HL-60 cells. DNA oligonucleotides complementary to LacZ (LacZ) and beads only (BO) were utilized as controls. RNA pull-down was performed with streptavidin-linked beads. The recovered linc-223 assessed by RT-PCR from input extract and pull-down with L223, lacZ and BO are shown. (**E**) Western blot analysis of Ago2 and HPRT from input, L223, LacZ and BO. (**F**) The histograms show the recovery of miR-125b-5p and miR-223 indicated as percentage of input in fraction bound to L223, lacZ and BO. Error bars represent S.E.M from triplicates. Input represents 10% of the extract used for the pull-down.

It is known that lncRNAs may regulate gene expression by several mechanisms. An intriguing possibility is that linc-223 can enter circuitries of gene expression regulation by acting as competing endogenous RNAs (ceRNAs) [17]. Bioinformatics analysis for miRNA binding sites on linc-223 identified the oncogenic miR-125-5p family (miR-125a, miR-125b1 and miR-125b2), which was shown to inhibit myeloid differentiation [18], and miR-223 itself as putative interactors. The miR-125-5p binding site is present in the third exon, which is in common to both isoforms, and it is conserved between human and mouse linc-223 despite a general low conservation between the linc-223 transcripts (Figure 4A). Thus, the linc-223 sequence (RLuc-linc223) and mutant derivatives lacking the putative miR-125-5p (RLuc-linc223-Δ125) or miR-223 (RLuc-linc223-Δ223) recognition sequences were cloned downstream of the luciferase gene and transfected together with either miR-125b or miR-223 coding plasmids (Figure 4B). The linc-223 sequence utilized in the reporter plasmids cannot produce miR-223 because is deleted for the region required for Drosha cleavage, but it still contained the region that can potentially base pair with miR-223. Luciferase expression of the RLuc-linc223 construct was significantly reduced with respect to the control plasmid (pCTR) when miR-125b was expressed (Figure 4C). Notably, repression was abolished when the mutant miR-125-5p derivative RLuc-linc223-Δ125 was utilized. Conversely, no reduction of luciferase activity was observed in the presence of miR-223 (Figure 4C). Thus, these data indicate that linc-223 can be specifically targeted by miR-125-5p.

In order to further analyze the interaction between linc-223 and miR-125-5p, we performed a RNA pull down assay against the endogenous linc-223 in AML cells by using biotinylated DNA oligonucleotides complementary to linc-223 with HL-60 cytoplasmic extract. Biotinylated DNA oligonucleotides complementary to LacZ were utilized as control. Notably, miR-125-5p and Ago2 protein were specifically recovered with linc-223 from the fraction bound to the specific probes (Figures 4D, 4E and 4F). The absence of miR-223 from the pull-down confirmed the specificity of the interaction between miR-125-5p and linc-223 (Figure 4F). Altogether these data indicate that linc-223 can interact *in vivo* with miR-125-5p, associated with the Ago protein, and support the hypothesis that linc-223 might act as a competing endogenous RNA (ceRNA) for miR-125-5p.

**Figure 5:**
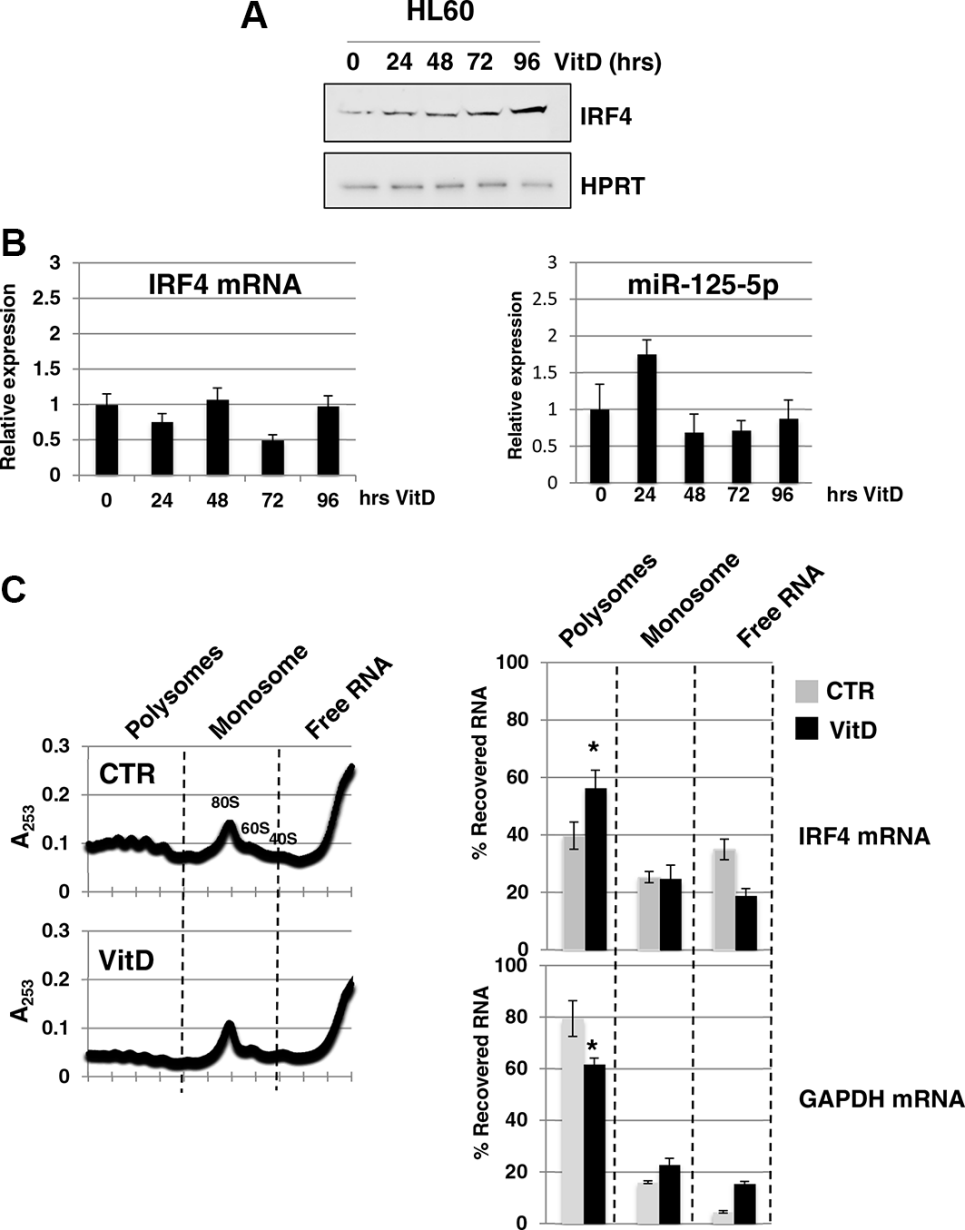
Analysis of IRF4 expression (**A**) Western blot analysis of IRF4 and HPRT protein levels during VitD3-induced monocytic differentiation of HL-60 cells. (**B**) qPCR analysis of IRF4 mRNA and miR-125b-5p levels during VitD3-induced monocytic differentiation of HL-60 cell line. Values were normalized for HPRT mRNA. (**C**) Cytoplasmic extracts from HL-60 cells, either untreated (CTR) or treated with VitD for 72 hours (VitD), were loaded on 15–50% sucrose gradients and fractions measured by absorbance at 253 nm. Fraction density decreases from left to right; the panels show one representative profile out of three independent biological replicates. Pooled fractions of RNA associated with polysomes, monosome and free RNA were analyzed by qRT-PCR and represented as percentage of RNA level in each of the fractions. Profiles are shown for IRF4 mRNA and GAPDH mRNAs.

MiR-125-5p is an oncogenic miRNA family that is able to impair myeloid differentiation and to transform myeloid cells [18, 19]. Among the validated miR-125-5p target mRNAs, we focused on the transcription factor IRF4 (interferon regulatory factor 4). Indeed, it has been shown that miR-125-5p induces tumorigenesis in myeloid cells through the repression of IRF4 mRNA [20] and, above all, that the expression of a IRF4 derivative without miR-125-5p binding sites rescues miR-125-5p induced myeloid leukemia [20]. IRF4 protein levels matches that ones of linc-223 during monocytic differentiation showing an increase between 72 hrs and 96 hrs of VitD treatment (Figure 5A). Furthermore, IRF4 mRNA and miR-125-5p levels did not change significantly during this period of time (Figure 5B). To better understand the regulation of IRF4 expression we performed polysome profile by sucrose gradient analysis of HL-60 cells, either untreated (CTR) or treated with VitD for 72 hours (VitD). Interestingly, the increase of IRF4 protein levels upon VitD treatment was accompanied by a shift of IRF4 mRNAs toward the polysomal fraction of the gradient (Figure 5C), despite a general decrease of translation. These data are consistent with a reduction of miRNA repressing activity on IRF4 mRNA.

**Figure 6:**
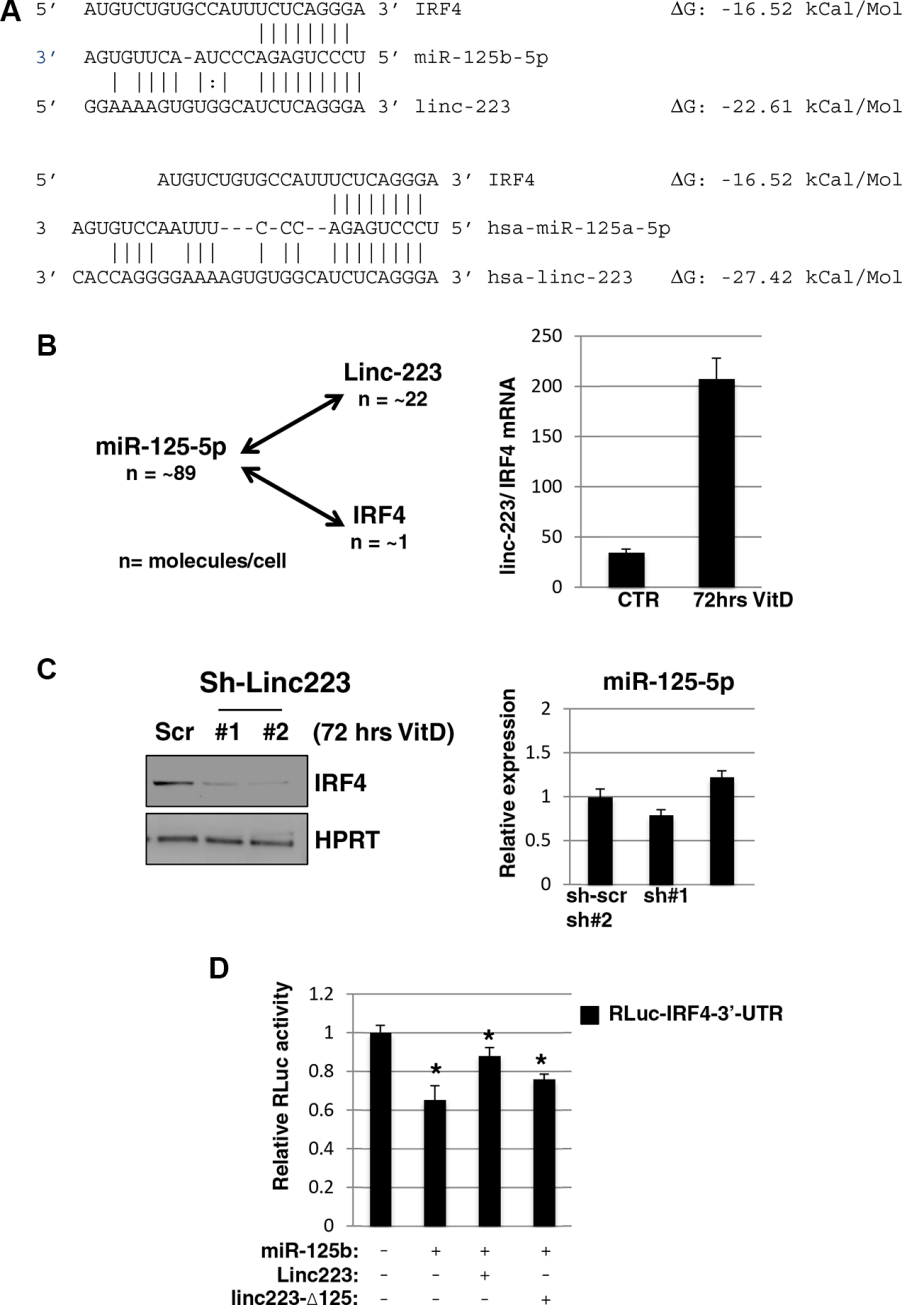
Linc-223 cross-talks with IRF4 (**A**) Base-pairing between miR-125-5p and linc-223, and miR-125-5p and IRF4 3′-UTR, ΔG values were obtained from miRanda (http://www.microrna.org). (**B**) Left panel: absolute number of miR-125-5p, linc-223 and IRF4 molecules for cells (n). Right panel: ratio between linc-223 and IRF-4 mRNA in HL-60, either untreated (CTR) or treated with VitD for 72 hours (VitD), measured by qPCR. Values were normalized for HPRT mRNA expression. (**C**) Left panel: western blot analysis of IRF4 and HPRT protein levels in HL-60 cells expressing scramble shRNA (sh-scr) or shRNAs against linc-223 (sh#1 and sh#2); right panel: qPCR analysis of miR-125b levels relative to U6 snRNA in HL-60 cells expressing scramble sh-scr or sh#1 and sh#2. (**D**) K562 cells were transfected with IRF4-3′UTR reporter together with plasmids expressing miR-125b-5p, RLuc-linc223 or RLuc-linc223-Δ125. FF luciferase values were normalized to RL luciferase reading. Error bars represent S.E.M from triplicates. Expression differences were statistically analyzed; *p* < 0.05 = *.

In line with a decoy mechanism, the predicted ΔG of binding of miR-125-5p with linc-223 is lower than that with IRF4 (Figure 6A). Absolute quantification in HL-60 indicated that miR-125-5p and linc-223 show comparable expression levels while linc-223 copies are about twenty times more then IRF4 (Supplemental Figure S4). Moreover, upon VitD treatment the expression of linc-223 is about 200 times that one of IRF4 while miR-125-5p levels are not changed significantly (Figures 5B and 6B). Therein, we analyzed the levels of IRF4 protein upon knock-down of linc-223 during VitD3-mediated monocytic differentiation. In agreement with our ceRNA hypothesis, we observed a significant reduction of IRF4 with lower linc-223 levels while miR-125b levels were not affected (Figure 6C). To further demonstrate the cross-talking between linc-223 and IRF-4, we transfected K562 cells with a luciferase reporter containing IRF4 3′-UTR (RLuc-IRF4-3′-UTR) in combination with miR-125b alone or together with wild-type linc-223 or a mutant derivative lacking the putative miR-125-5p binding site (linc223-Δ125), respectively. As expected, miR-125b decreased the expression of RLuc-IRF4-3′-UTR (Figure 6D). The concomitant expression of linc-223 partially recovered the IRF4 repression while the expression of linc223-Δ125 showed a minor but still significant effect. These data indicate the existence of a specific crosstalk between linc-223 and IRF4 mRNA through competition for miR-125-5p binding.

**Figure 7:**
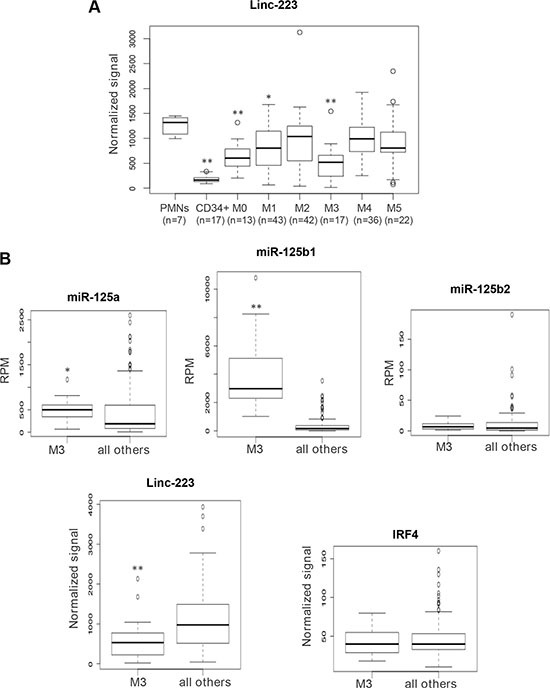
Analysis of linc-223 expression in AML patients and normal hematopoietic cells (**A**) Quantitative expression analysis of linc-223 in primary leukemia cells derived from AML patients from TCGA, normal CD34+ progenitors and mature polymorphonucleated cells (PMNs). The different FAB subtypes are indicated in the graph. (**B**) Quantitative expression analysis of miR-125-5p family members, linc-223 and IRF4 in AML of the M3 subtype (M3) compared to all the other subtypes (all other). Expression differences were statistically analyzed; *p* < 0.01 *; *p* < 0.001**

### Linc-223 is downregulated in AML

We evaluated linc-223 expression in gene expression arrays of different AML subtypes (Supplementary Table S1), mature polymorphonucleated cells (PMNs) and CD34+ progenitor cells. We found significant downregulation of linc-223 expression compared to PMNs in AML of the M0, M1 and M3 subtypes (Figure 7A). The lowest expression of linc-223 was detected in AML of the M3 subtype. Notably, although miR-125-5p is able to induce leukemia in mouse when overexpressed in the hematopoietic system [18, 21], in primary human AML it was found upregulated only in the M3 FAB subtype [22, 23]. Thus, we analyzed the correlation of miR-125-5p family members (miR-125a, miR-125b1 and miR-125b2), IRF4 and linc-223 in AML M3 with respect to all others AML subtypes. As reported, we observed a specific upregulation of miR-125b in AML-M3. Interestingly, an inverse correlation between the expressions of miR-125b and linc-223 was observed while IRF4 expression was constant (Figure 7B). Therein, these data indicate that the linc-223 downregulation observed in AML M3 might contribute to the oncogenic activity of miR-125-5p by increasing its repressing activity on IRF4 mRNA.

## DISCUSSION

Several lncRNAs have been shown to be involved in normal and malignant hematopoiesis [2]. However, only few lncRNAs have been functionally characterized. In recent years, it has been discovered that lncRNAs can prevent miRNA repressive activity on their target mRNAs through binding to specific miRNAs [12]. Thus, acting as competing endogenous RNAs (ceRNAs). Several studies showed that the disruption of the equilibrium between ceRNAs and miRNAs can be critical in many biological processes and can contribute to cancer development [17, 24–6]. In this manuscript, we show that the host non-coding transcript of miR-223, linc-223, is a functional lncRNA that controls proliferation and differentiation of AML cells, at least in part, by binding miR-125-5p and competing for its repressing activity on IRF4. MiR-125-5p has been previously associated with leukemogenesis [27]. In particular, MiR-125b was found significantly increased in primary AML cells of the M3 FAB subtype [22, 23] where linc-223 was instead found at low expression levels. MiR-125b is able to induce myeloid leukemia when overexpressed in the hematopoietic compartment in mouse [18, 21]. Notably, the oncogenic activity of miR-125b *in vivo* can be rescued by the expression of its target IRF4 devoid of miR-125b binding sites [20]. Therein, indicating IRF4 as a crucial miR-125-5p target in myeloid leukemia. Here, we show that decrease in linc-223 levels produce a downregulation of IRF4 expression, thus pointing to linc-223 as a novel oncosuppressor in AML.

As linc-223 and IRF4 might share binding sites for other different microRNAs, we can speculate that their cross-talking might be much more complex than what has been described in this study. Furthermore, the regulative network described here might represent a small part of a more elaborated cross-talk, which might include many other components (such us pseudogenes, circRNAs and other lncRNAs).

Whether linc-223 affects miR-125-5p function *in vivo* requires to be further investigated and represents a relevant topic to be addressed in the future.

Interestingly, we observed different regulation of miR-223 and linc-223 expression during monocytic differentiation of both normal hematopoietic progenitors and AML cells. MiR-223 transcription is predominantly regulated by a conserved promoter in front of the pri-miR-223/linc-223 transcription start site [7, 28] and, to a lesser extent, by a non-conserved intronic promoter upstream to the pre-miRNA sequence [6, 28, 14]. Previous data showed that both promoters determine the high expression levels of mature miR-223 in granulocytic cells. On the other hand, pri-miR-223/linc-223 showed different expression pattern relative to miR-223 during monocytic differentiation. Moreover, while miR-223 expression promotes granulocytic differentiation it impairs monocytopoieis of CD34+ [14]. Therefore, the balance between linc-223 and miR-223 expression may be relevant for the lineage-specific differentiation/maturation choice of myeloid progenitors during hematopoiesis. Our data suggest that during monocytic differentiation a specific post-transcriptional mechanism could control the relative production of linc-223 versus miR-223, possibly at the level of microprocessor activity, similarly to what already shown for other miRNA-containing lncRNAs [29, 30]. Further studies are needed to unravel these mechanisms and to determine their implications in AML.

## MATERIALS AND METHODS

### Cell cultures and reagents

HL-60 and K562 cell lines were maintained in RPMI 1640 medium supplemented with 1 × Penicillin/Streptomicin solution, 1 × L-glutamine and 10% Fetal Bovine Serum. All utilized cell lines were tested for mycoplasma contamination. All-trans-retinoic acid (ATRA) and 1,25-Dihydroxyvitamin D3 (VitD) were purchased from Sigma and utilized at a concentration of 1 μM and 250 ng/ml, respectively, unless differently specified. Doxycycline (Dox) was purchased from Sigma and utilized at a concentration of 200 ng/ml, unless differently specified.

### RNA extraction and real-time qRT-PCR analysis

Total RNA was extracted using the Quick RNA miniprep kit (Zymo) according to manufacturer instructions. For mRNA analysis, reverse transcription to cDNA was performed with the SuperScript VILO cDNA Synthesis Kit (Life Technologies) according to the manufacturer instructions. Quantitative real-time PCR was performed on an Applied Biosystems 7500 Fast Real Time PCR System. Reactions were performed in triplicate using the SYBR green dye detection system and analyzed using 7500 Software v2.0.6 (Applied Biosystems). Relative expression levels of targets were determined using the comparative 2^ΔΔCt^ method. Hypoxanthine-guanine phosphoribosyltransferase (HPRT) mRNA was utilized as a reference. MicroRNA analysis by real-time PCR was performed using miScript System (QIAGEN) for hsa-miR-223 and hsa-miR-125b, and delta-delta Ct values were normalized with those obtained from the amplification of the endogenous U6 snRNA (QIAGEN). All reactions were performed in triplicate. Expression differences were statistically analyzed using a Student's *t-test*.

Nuclear/cytoplasmic fractionation was carried out by using the NE-Per Kit (Thermo Scientific-Pierce) according to the manufacturer's specifications. PolyA+ RNA fraction was obtained using Oligotex mRNA Mini Kit (Qiagen) according to the manufacturer's specifications. RT-PCRs were performed by amplifying cDNA with MyTaq™ HS Red DNA Polymerase (Bioline) and specific oligonucleotides listed below.

Relative quantification of miR-125-5p, linc-223 and IRF4 mRNA was performed by real-time PCR using miScript II reverse transcription system (QIAGEN), and normalized with the endogenous HPRT. Absolute quantification of miR-125-5p, linc-223 and IRF4 was measured through qRT-PCR calibrating with an internal standard curve of synthetic constructs, as previously described [31].

### Linc-223 knockdown and overexpression

Linc-223 knockdown was obtained by Mission Lentiviral shRNA clones (Sigma-Aldrich, USA). Mission Lentiviral Non-Targeting shRNA clone SHC002 (Sigma-Aldrich, USA) was utilized as control. Lentiviral particles were prepared according to the manufacturer's specifications. Infection of AML cell lines was performed as previously described [32]. Targeting sequences are CTGTCCTAGAGAAACTTTATA for Sh#1 and CAGCCCAGTACTTTAGTTACA for Sh#2.

For linc-223 ectopic expression, stable and inducible HL-60 cell lines were produced as previously described [15, 16]. Briefly, linc-223 cDNA was amplified from HL-60 cells and subcloned in the enhanced PiggyBac (ePB) vector ePB-PURO using the oligonucleotides Linc-223-BamHI-FW and Linc-223-NotI-REV. Deletion of the Drosha cleavage site was obtains by inverse PCR with oligonucleotides Linc-223-mut.223-FW and Linc-223-mut.223-REV generating the EBP-linc223 plasmid. GFP sequence for control EBP was amplified from a commercial EBP plasmid contains a TET-on system for inducible transgene expression. Helper and transposon plasmids were electroporated in HL-60 with the Neon Transfection System (Invitrogen) according to manufacturer instruction. Selection with 1 μg/mL of puromycin (SIGMA) was initiated 2 days after transfection and maintained until resistant colonies became visible.

### RNA pull-down

HL-60 cells were treated with VitD for 72 hours and 30 × 10^6^ cells were collected and washed with ice cold PBS and resuspended in Buffer A (20 mM Tris pH 7.4, 10 mM NaCl, 3 mM MgCl, 0.1% NP40, 0.2 mM EDTA, 10% glycerol). 800 μg of supernatant (cytoplasmic extract) were incubated with tRNA-saturated magnetic beads (Streptavidin MagneSphere Paramagnetic Particles, Promega) and ten biotinylated DNA probes in IP-Buffer (20 mM Hepes KOH pH 7.4, 150 mM KCl, 2 mM MgCl2, 0.01% Triton X-100, 5% glycerol, 1 mM DTT) for 1 hrs at R.T. Beads were washed four times with IP-Buffer and resuspended in 1 mL of Trizol and Protein loading dye for RNA and protein analysis, respectively. Protein levels were analyzed by western blot as previously described (Hughes et al., 2015). Antibodies utilized were against Ago2 (ab32381, Abcam) and HPRT (FL-218, Santacruz Biotechnology).

### Luciferase assay

Linc-223 was amplified with oligonucleotides Linc-223-XhoI-FW and Linc-223-NotI-REV then cloned in the psicheck2 plasmid (Promega), downstream from the Renilla luciferase (RLuc) gene. The same plasmid also contains the Firefly luciferase (FLuc) to normalize transfection efficiency. The mutant derivative lacking the miR-125 binding site was obtained by inverse PCR with the oligonucleotides Linc-223-mut.125b-FW and Linc-223-mut.125b-REV. The mutant derivative lacking the miR-223 binding site was obtained by inverse PCR with the oligonucleotides Linc-223-mut.223-FW and Linc-223-mut.223-REV. IRF4 3′-UTR was amplified with oligonucleotides IRF4-XhoI-FW and IRF4-NotI-REV then cloned in the psicheck2 plasmid (Promega), downstream from the Renilla luciferase (RLuc) gene. RLuc and FLuc activities were measured by Dual Glo luciferase assay (Promega). Expression differences were statistically analyzed using a Student's *t-test*.

### Cell proliferation and differentiation

For cell cycle analysis, 2 × 10^5^ cells were resuspended in 50% FCS, fixed in 70% ethanol for 24 hours, incubated with 50 Ag/mL propidium iodide (Sigma-Aldrich) and 50 units/mL DNase free RNase A (Sigma-Aldrich), and analyzed after 3 hours (10,000 events) using an Epics XL Cytometer (Beckman Coulter). Differentiation was assessed by NBT dye reduction assay and direct immunofluorescence staining of cells using an Allophycocyanin (APC) anti-human CD11b (Becton Dickinson), APC anti-human PerCP-Cy5.5 anti-human CD14 (Becton Dickinson) and PE-IgG1 isotype control (eBiosciences) as previously described (Salvatori et al., 2012). A minimum of 10,000 events was collected for each sample with flow cytometer (CyAN ADP DAKO) by using Summit 4.3 software for data acquisition and analysis.

### Polysome profiling

Cytoplasm fractionations on sucrose gradients were performed as follows: 20 × 10^6^ cells were lysed with 500 ml of lysis buffer (10 mM Tris pH 7.5, 100 mM NaCl, 10 mM MgCl_2_, 0.5% Triton X-100, and 0.5% sodium deoxycholate) supplemented with 100 mg/ml cycloheximide, 1X PIC (Complete, EDTA free, Roche) and 1X RNase guard (Thermo Scientific). The lysates were centrifuged for 10 min at 13000 rpm at 4°C. The supernatants were collected and centrifuged on 15%–45% sucrose gradient at 38000 rpm with a SW41 rotor (Beckman) for 1 h 30 min at 4°C. Fractions were collected with a Bio-logic LP (Biorad), 10 mg of glycogen and 100 μg of Proteinase K (Roche) was added to each fraction. Samples were left 1 h at 37°C and RNA was purified by PCA extraction and precipitated with isopropanol.

### Data analysis

Data were downloaded from GEO DataSets GSE16020 (PMN_control_Rneasy 1–7), GSE19429 (CD34+ Healthy control 1–17) and from the TCGA Research Network (AML samples). Information of TCGA AML patients are listed in Supplementary Table S1. All data were derived from microarray experiments performed with GeneChip^®^ Human Genome U133 Plus 2.0 Array (GPL570). We used the software package DNA-Chip Analyzer (dChip) for probe-level (e.g. Affymetrix platform) and high-level analysis of gene expression microarrays and SNP microarrays to normalize CEL files. The normalization was performed using an array with median overall intensity chosen as the baseline array against which other arrays are normalized at probe intensity level. In this way the brightness of the arrays was adjusted to comparable level. We utilized the same software to compute model-based expression values for each arrays. The expression values of interest were than analyzed. *P* value was calculated using two-tailed Mann-Whitney *U*-test.

**Table:** 

Oligonucleotides	
Oligonucleotides used in this study:	
*qRT-PCR:*	
Linc-223 FW	GAAAGCCCAATTCCATCTG
Linc-223 REV	AGTGGAGTGGTGCCTTGGT
MCSFR FW	TCCAAAACACGGGGACCTATC
MCSFR REV	TCCTCGAACACGACCACCT
IRF4-FW	CAGTTCAGCGGTTGAGGAGA
IRF4-REV	AATGCAAAGCCACCCTTCCT
CD11b-FW	CCTGGTGTTCTTGGTGCCC
CD11b-REV	TCCTTGGTGTGGCACGTACTC
CD14-FW	AAAGGACTGCCAGCCAAGCT
CD14-REV	GATTCCCGTCCAGTGTCAGGT
*RT-PCR:*	
Linc-223 FW	CAAGGCACCACTCCACTGAC
Linc-223 REV	ATATCCCATCTGCCCTGGC
Pri-miR-223 FW	TCAGGATCTCTCTTCTGGTTAGG
Pri-miR-223 REV	GATCTTGCTCAAAGGACCAAC
Linc-223-2 FW	ACTGACAGGGTCACATCT
Linc-223-2 REV	GACAAACTGACACTCTACCAC
HPRT FW	GCCATCACATTGTAGCCCTCTG
HPRT REV	TTTATGTCCCCTGTTGACTGGTC
*Cloning:*	
Linc-223 BamHI FW	CGCGGATCCAGAAAGCCCAATTCCATC
Linc-223 NotI REV	AAAAGCGGCCGCAACTCCTCAGTGCATATATTTATT
Linc-223-mut.223 FW	AAGTGCGGCACATGCTTACCA
Linc-223-mut.223 REV	CTCTACCACATGGAGTGTCCAA
Linc-223-mut.125b FW	AAATACAGCCCTGGGCTGTG
Linc-223-mut.125b REV	ATGCCACACTTTTCCCCTGG
Linc-223 XhoI FW	CCGCTCGAGAGAAAGCCCAATTCCATCTGGCCC
IRF4 XhoI FW	CCGCTCGAGAAAATGTCAAGATGAGTGGTT
IRF4 NotI REV	AAAAGCGGCCGCTAACAAATTTTTAAAAGTTTTTAT
*Linc-223 pull down:*	
Oligo#1	CATTCGTCATATCCCATCTG
Oligo#2	GGTGTCCTCAGATATAATGC
Oligo#3	GAGAATGCTGATGTCCTAGA
Oligo#4	CTTTAGCTGGTCCAGGAAAT
Oligo#5	ATCCTTCATGCTGGCTACTA
Oligo#6	AACTTGTGAGAACTTGGTGC
Oligo#7	TCATGGTGTGTGTAGACACA
Oligo#8	CAGATAAGCCATTAGGCTTG
Oligo#9	GGTGCTATTAGACAGTAGGA
Oligo#10	GACACTGTTTCTCAGGAAGA
*LacZ pull down:*	
Oligo#1	CCAGTGAATCCGTAATCA
Oligo#2	GTAGCCAGCTTTCATCAACA
Oligo#3	AATGTGAGCGAGTAACAACC
Oligo#4	AATAATTCGCGTCTGGCCTT
Oligo#5	ATTAAGTTGGGTAACGCCAG
Oligo#6	AATTCAGACGGCAAACGACT
Oligo#7	AATAATTCGCGTCTGGCCTT
Oligo#8	AGATGAAACGCCGAGTTAAC
Oligo#9	AATTCAGACGGCAAACGCT
Oligo#10	TTTCTCCGGCGCGTAAAAAT

## SUPPLEMENTARY MATERIALS FIGURES AND TABLE




