# Supplementary material for: The miR-223 host non-coding transcript linc-223 induces IRF4 expression in acute myeloid leukemia by acting as a competing endogenous RNA
Source: Oncotarget. 2016 Aug 9;7(37):60155–68. doi: 10.18632/oncotarget.11165 (PMC5312375; doi:10.18632/oncotarget.11165)
Supplement: Supplementary file 1 [file oncotarget-07-60155-s001.pdf]

The miR-223 host non-coding transcript linc-223 induces IRF4 expression in acute myeloid leukemia by acting as a competing endogenous RNA

Supplementary Materials

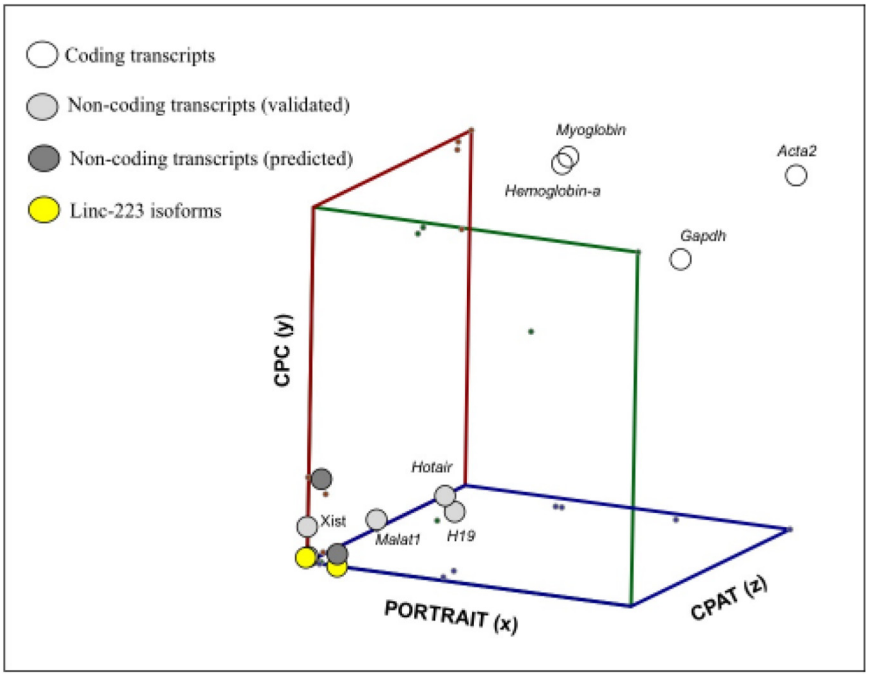

Supplementary Figure S1: Bioinformatics prediction of linc-223 coding potential. We used three different tools to predict the coding potential of transcripts above: CPC (Coding Potential Calculator) (cpc.cbi.pku.edu.cn), CPAT (Coding-Potential Assessment Tool) (code.google.com/p/cpat/) and PORTRAIT (bioinformatics.cenargen.embrapa.br/portrait/). The crossed results can be visualized in a scatter plot. Known coding transcripts (white circles), such as the mRNA of GAPDH or HEMOGLOBIN-A, localize in the upper right of the plot because of their high score. Conversely, the low score of non-coding transcripts (in light and dark gray), such as lncRNAs XIST or HOTAIR, placed them in the bottom left of the plot. According to this chart, Linc-223 alternative isoforms (yellow circles) have a very low probability to be coding.

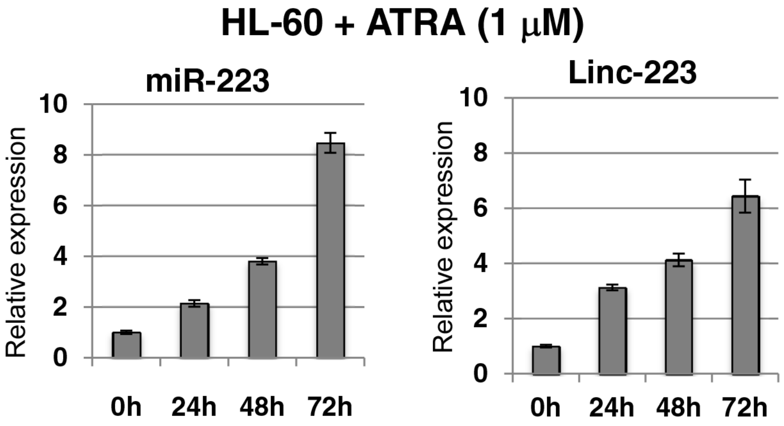

Supplementary Figure S2: Linc-223 expression levels increase during ATRA-mediated granulocytic differentiation. qPCR analysis of miR-223 and linc-223 levels during ATRA-induced granulocytic differentiation of HL-60 cell line. Values were normalized for U6 and HPRT mRNA expression, respectively. The histograms represent the means ± S.E.M. from triplicates.

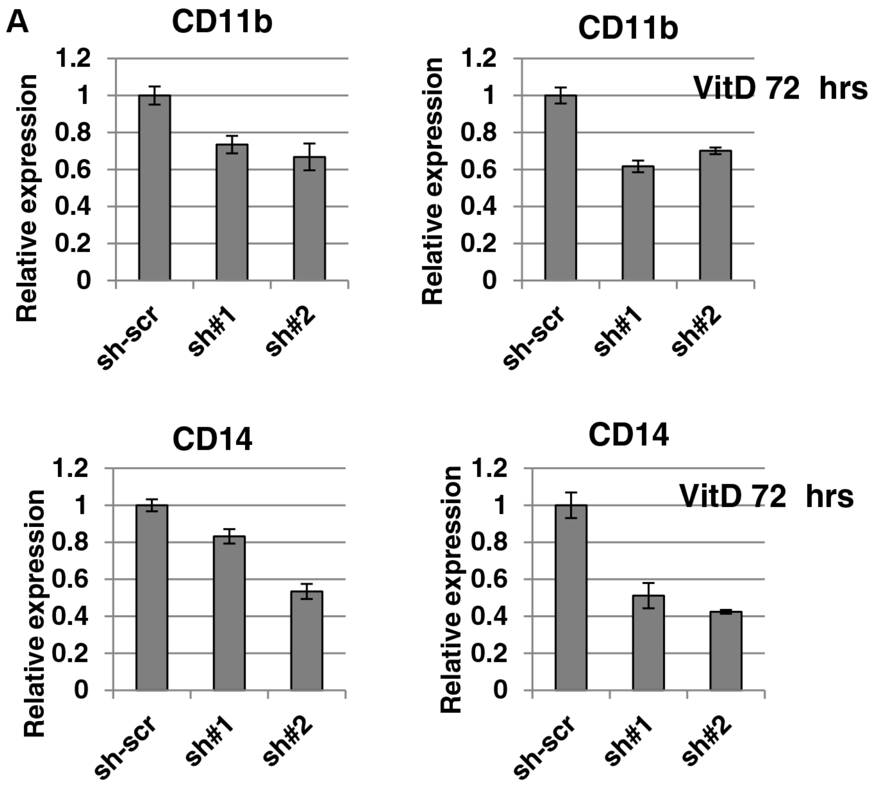

Supplementary Figure S3: qPCR analysis od CD11b and CD14 in HL-60 cells expressing scramble shRNA or shRNAs againsts linc-223 in untreated (left panels) or 72 hours of VitD3 treatment (right panels). Error bars represent S.E.M. from three independent experiments.

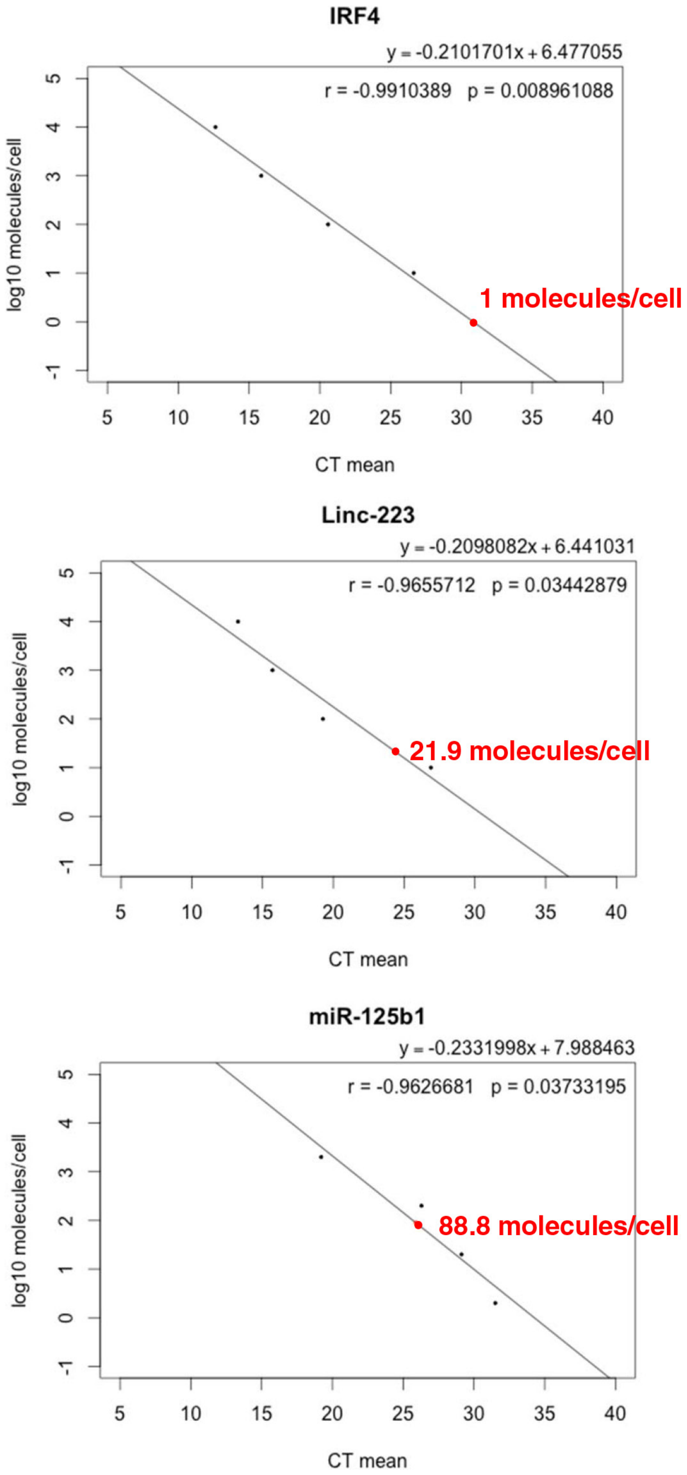

Supplementary Figure S4: Absolute quantification of IRF4, linc-223 and miR-125-5p in HL-60 cells was measured with an internal standard curve of synthetic constructs.

Supplementary Table S1: Clinical data of AML patients.
